# Morphology, structural properties and reducibility of size-selected CeO_2−_*_x_* nanoparticle films

**DOI:** 10.3762/bjnano.6.7

**Published:** 2015-01-07

**Authors:** Maria Chiara Spadaro, Sergio D’Addato, Gabriele Gasperi, Francesco Benedetti, Paola Luches, Vincenzo Grillo, Giovanni Bertoni, Sergio Valeri

**Affiliations:** 1 CNR-NANO, Centro di Ricerca S3, via G. Campi 213/a, Modena, Italy; 2 Dipartimento FIM, Università di Modena e Reggio Emilia, Via G. Campi 213/a, Modena, Italy; 3 CNR-IMEM, Parco Area delle Scienze 37/A - 43124 Parma, Italy

**Keywords:** CeO_2_ ultra-thin films, ceria nanoparticles, magnetron sputtering, reduction and oxidation, size-dependent properties, size-selected nanoparticles, X-ray photoelectron spectroscopy

## Abstract

Non-stoichiometric ceria nanoparticles (NPs) were obtained by a gas aggregation source with a magnetron and were mass-selected with a quadrupole mass filter. By varying magnetron power, Ar gas flow, and the length of the aggregation tube, NPs with an average diameter of 6, 9, and 14 nm were synthesized and deposited onto a substrate, thus obtaining NP films. The morphology of the films was studied with scanning electron microscopy, while high resolution transmission electron microscopy was used to gain a deeper insight into the atomic structure of individual NPs. By using X-ray photoelectron spectroscopy we analyzed the degree of reduction of the NPs of different diameters, before and after thermal treatments in vacuum (reduction cycle) and in O_2_ atmosphere (oxidation cycle) at different temperatures. From this analysis we inferred that the size is an important parameter only at intermediate temperatures. As a comparison, we evaluated the reducibility of an ultra-thin ceria film with the same surface to volume ratio as the 9 nm diameter NPs film, observing that NPs are more reducible than the ceria film.

## Introduction

The main property of cerium oxide that attracts scientific attention is its ability to store and release oxygen depending on the ambient conditions [1]. In particular, ceria in the form of nanoparticles (NPs) is important in industrial catalysis [2] and in biomedical applications to prevent the oxidation of human cells [3]. Doped cerium oxide films are also promising candidates as electrolytes in solid oxide fuel cells [4].

A lot of studies have been performed on ceria NPs while varying their diameter: NPs with diameter less than 5 nm have larger oxygen storage capacity than the ones with higher diameter; this is related to the larger surface area exposed by the smaller NPs [5].

It is well known that in CeO_2−_*_x_* NPs the lattice parameter increases when the particle size is decreased. Tsunekawa et al. [6], analyzing NPs with diameter between 2 nm and 4 nm, suggested that the reduction of the Ce ion charge from 4+ to 3+ leads to an increase of the lattice parameter because of the decrease in the electrostatic force. With the assumption that the increase of the lattice parameter is also due to a higher concentration of oxygen vacancies, Tsunekawa results are complementary with the ones of Zhou et al. [7], obtained for NP diameters between 4 and 60 nm. These results led to the conclusion that the lattice parameter increase is related to the formation of oxygen vacancies and Ce^3+^ ions.

Following this approach, Deshpande et al. [8] correlated the lattice parameter expansion with the concentration of Ce^3+^ ions (measured by X-ray photoelectron spectroscopy, XPS), ascribing it to the higher ionic radius of Ce^3+^, compared to the Ce^4+^, and to the introduction of oxygen vacancies, which in turn induces a distortion of the local symmetry. In the last years a ‘Madelung model’ has been proposed to describe the properties of ionic crystals as a function of their surface to volume ratio. Here, the balance between long range Coulomb attractive and short range repulsive interactions is broken, leading to an effective negative pressure and thus to an increase of the lattice parameter [9]. Actually, a proper combination of all these factors, namely the increase of concentration of Ce^3+^ ions, oxygen vacancies and Madelung pressure, can explain the observed phenomena.

It is not yet clear if the presence of Ce^3+^ ions is an intrinsic characteristic of the NP [10] or if it is related to the synthesis procedure. Paun et al. [11] synthesized ceria NPs with different diameters and identical polyhedral shapes, by means of different chemical synthesis procedures. The concentration of Ce^3+^ ions was found to be quite different even for NPs with the same diameter, showing that the presence of Ce^3+^ ions is also related to the synthesis procedure and not only to the particle size.

Few works have been performed with NPs synthetized by magnetron sputtering, the technique used in this study. Tschöpe et al. [12] studied ceria NPs realized by magnetron sputtering from pure and mixed metal target and inert gas condensation, observing the high non-stoichiometry of these systems due to the particular synthesis method. The non-stoichiometry is due to the presence of Ce^3+^ ions. Non-stoichiometric NPs grown in this way exhibit a higher catalytic activity than stoichiometric material, mainly because of surface defects and chemisorbed oxygen [13–14]. A new interpretation for the redox activity of CeO_2−_*_x_* NPs has been recently proposed, based on the increase of electron density in delocalized mixed cerium and oxygen orbitals, rather than on localized surface reduction of Ce^4+^ to Ce^3+^ ionic species [15].

In this work we present the results of the study of CeO_2−_*_x_* NPs produced by combining magnetron sputtering with a gas aggregation source. We investigated NPs reducibility as a function of their diameter (ranging from 6 to 14 nm) under reduction and oxidation conditions, and in comparison with a ultra-thin ceria film of the same surface to volume ratio as the 9 nm diameter NPs film. The NPs have been characterized with regard to morphology and structure by scanning electron microscopy (SEM) and high resolution transmission electron microscopy (HRTEM). The thermal stability of the NPs was investigated by XPS. The aim of this work is to investigate the fundamental relationship between NPs chemical and physical properties, in order to improve the understanding of the basic processes, which are fundamental for the ceria NPs applications.

## Experimental

**Figure 1:**
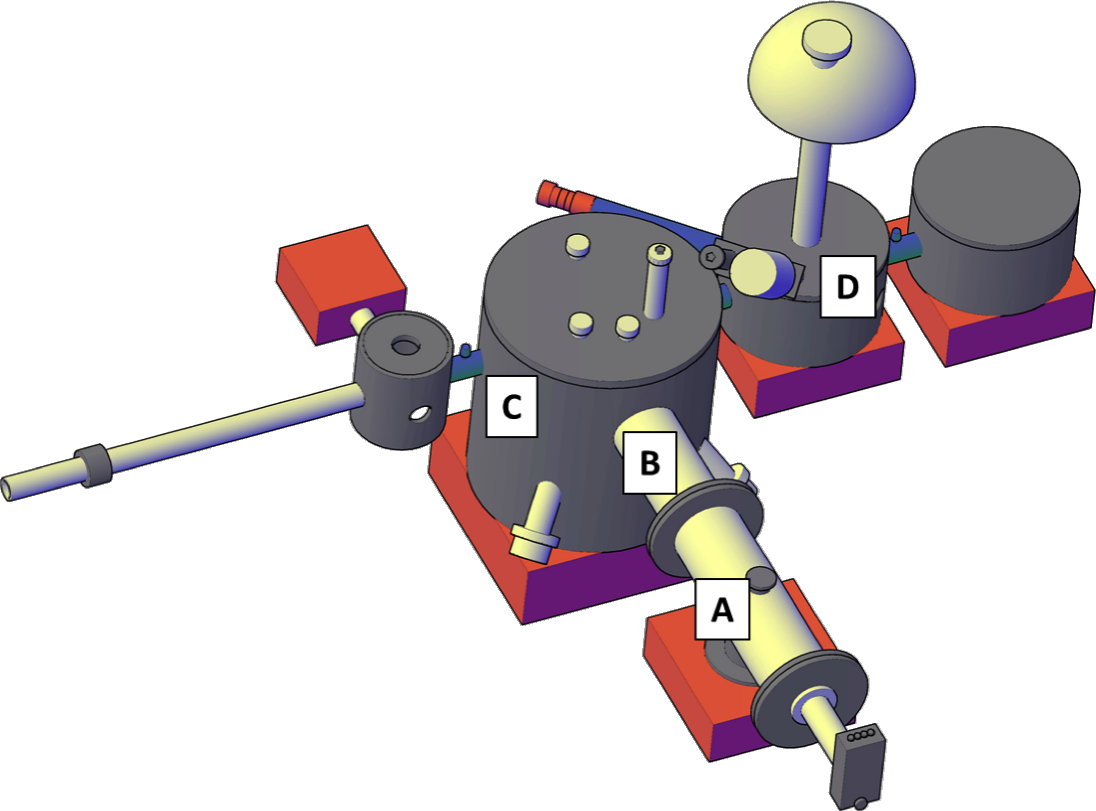
Sketch of the experimental set up: the NPs are created by the NC200 source (A), they are mass selected by the QMF (B) and they are deposited on the substrate in the deposition chamber (C). The chemical characterization is performed in situ in the XPS chamber (D).

The ceria NPs were synthesized at the SESAMo Laboratory [16–18], with an experimental system composed of three interconnected vacuum chambers (schematic view in Figure 1); the deposition chamber (C) is connected on one side to the NPs source chamber (A), equipped also with a quadrupole mass filter (B), and on the other side to the XPS chamber (D) for in situ chemical characterization. Ceria NPs were obtained by DC magnetron sputtering and inert gas (Ar) aggregation method, from a pure Ce target (99.9%).

Most of the clusters were charged, so that they could be mass selected by the quadrupole mass filter (QMF). The NP beam reached the deposition chamber, where a NP film was grown on a Si/SiO*_x_* substrate. Depositions were carried out in oxygen atmosphere (*p*_O2_ = 5·10^−6^ mbar) and post-oxidation for 30 min (*p*_O2_ = 4·10^−5^ mbar), to fully oxidize the NPs. The nominal film thickness was fixed to 10 nm (the evaporation rate was measured by a quartz microbalance in the deposition chamber) for all samples described in this work. The amount values of deposited NPs are given in terms of nominal thickness of an equivalent continuous film with the same density as CeO_2_. By changing the length of the aggregation tube, the electrical power applied to the target and the Ar gas flux, we obtained NPs with different average lateral sizes: 6, 9 and 14 nm.

In particular the magnetron discharge power (*P*), Ar flux (*f*) and aggregation length conditions (*l*) used were: 1) 6 nm NPs: *P* = 67 W, *f* = 10 sccm, *l* = 50 mm; 2) 9 nm NPs: *P* = 30 W, *f* = 40 sccm; *l* = 50 mm; 3) 14 nm NPs: *P* = 99 W, *f* = 56 sccm, *l* = 150 mm. The NPs diameter is controlled by the QMF monitor during the deposition, in fact it is possible to check the NPs diameter distribution scanning the quadrupole voltage whilst monitoring the NPs beam ion current. We also check the diameter distribution by ex situ SEM measurements, with a dual beam system (FEI Strata DB235M), in order to perform a statistical analysis and to have information on the mean diameter value and the full width at half maximum (FWHM) of the size distribution.

After deposition the samples were analyzed with in situ XPS, by using a twin anode X-ray source (XR50, Specs), generating Al Kα photons and a hemispherical electron analyzer (Phoibos 150, Specs). The reduction and oxidation cycles were performed in a different UHV apparatus, described in the second part of this section. After transferring the sample to the second apparatus the Ce^3+^ concentration in the NPs slightly increased from its original value measured immediately after growth, because of air exposure (almost 4% for all samples). We do not expect this modification to affect the oxygen transport in the NPs. For the reduction process the samples were heated in UHV at *T* = 520 K, *T* = 770 K and *T* = 1020 K for 30 min; for the oxidation process the samples were heated at *T* = 1020 K in O_2_ (*p* = 10^−7^ mbar) for 30 min. To estimate the contribution to the XPS spectra due to Ce^3+^ and Ce^4+^ ions we performed a fit with a linear combination of the Ce^3+^ and Ce^4+^ reference spectra, and by using the fitting equation

[1]
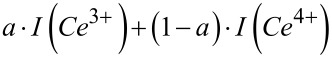


From the fit we evaluated the parameter *a*, which represents an estimate of the Ce^3+^ concentration. The error was estimated through the fitting procedure.

The Ce^3+^ and Ce^4+^ reference spectra were obtained from NP films treated with the following procedures:

To obtain the Ce^4+^ reference sample the deposited NPs were oxidized during their growth, injecting oxygen in the aggregation chamber (A region in Figure 1). The obtained NPs had an average lateral size <*d>* = 9 nm. A post-annealing at 1020 K in a rich oxygen atmosphere (10^−7^ mbar) was also performed. The reference spectrum for the reduced component *I*(Ce^3^*^+^*) was obtained from a film of NPs with <*d*> = 9 nm grown under high vacuum conditions, without the presence of oxygen. The ’as deposited’ sample was then annealed at *T* = 1020 K in UHV.

HRTEM experiments were performed by using a JEOL JEM-2200FS instrument working at 200 keV and equipped with a Schottky emitter. The instrument has an objective lens spherical aberration coefficient of 0.5 mm, providing a point-to-point resolution of 0.19 nm. The images were subsequently elaborated by using the STEM_CELL software [19]. Concerning the ultra-thin films we evaluated the morphology with in situ STM measurements by using an OMICRON room temperature SPM. The STM images have been processed by using the Image SXM software [20].

A second UHV apparatus was used to grow both epitaxial and non-epitaxial cerium oxide ultrathin films for comparison. The system is equipped with facilities for substrate preparation, film growth, in situ XPS, and scanning tunnelling microscopy (STM) analysis. The substrate used for film growth was a Pt(111) single crystal prepared by repeated cycles of sputtering (1 keV, 1 μA) and annealing (1040 K).

A 2 ML cerium oxide epitaxial film with the same surface-to-volume ratio of the NPs with 9 nm diameter (*S*/*V* = 0.6 nm^−1^), was grown with the procedures described in [21], i.e., reactive Ce electron-beam deposition in *p*_O2_ = 1·10^−7^ mbar at room temperature and post-growth annealing at *T* = 1040 K under the same O_2_ partial pressure. A non-epitaxial cerium oxide film grown on Pt(111) with nominal thickness *t* = 2 ML, was obtained with the same procedures as the epitaxial film, without the post-growth annealing in O_2_, and it was studied for further comparison.

Reducing thermal treatments were performed by using an electron bombardment heater. The samples were heated in UHV to the desired temperature, kept at that temperature for 30 min, and cooled to room temperature, following [22]. The oxidizing thermal treatments were performed under an oxygen partial pressure of *p*_O2_ = 1·10^−7^ mbar. XPS measurements were performed using an Al Kα X-ray source and a hemispherical electron analyzer. The STM was operated at room temperature in constant current mode, by using electrochemically etched tungsten tips, degassed by thermal treatments and sputtered by ion bombardment before the measurements.

## Results and Discussion

**Figure 2:**
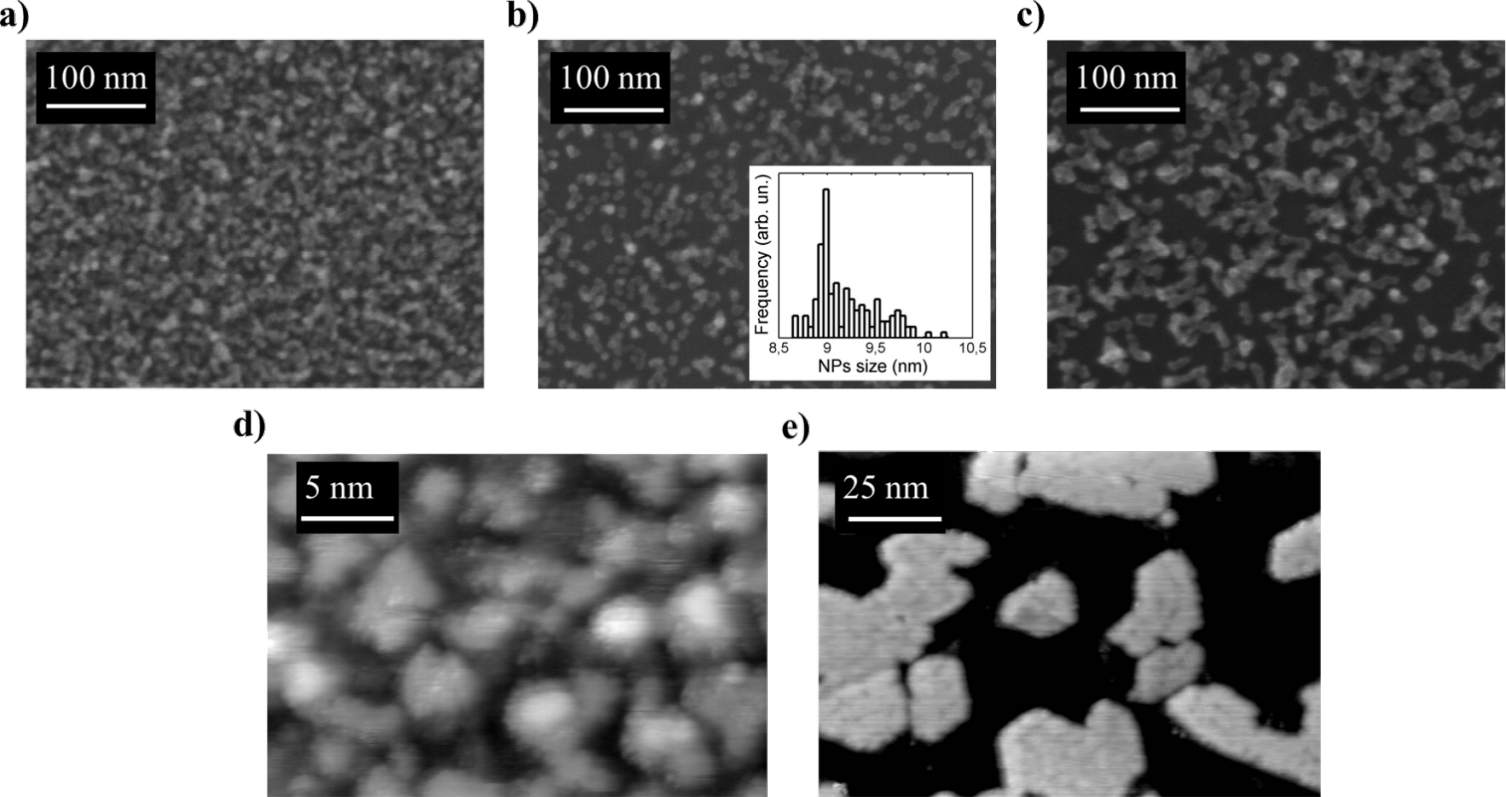
SEM images of NPs films: NPs diameter 6 nm (a), NPs diameter 9 nm (lateral size distribution in the inset) (b) and NPs diameter 14 nm (c). STM images of the non-epitaxial (d) and epitaxial (e) ultra-thin ceria films acquired at 1.5 V and 0.04 nA.

Figure 2 shows SEM images of NPs with different size values, and STM images of the epitaxial and non-epitaxial film. The 9 nm NPs (Figure 2b) are clearly visible and well dispersed, and it was possible to obtain the lateral size distribution shown in Figure 2b. The size distribution was obtained by measuring the area of 125 different NPs from SEM images and by evaluating the corresponding diameters, which range between 8.5 nm and 10.5 nm. By fitting the diameter histogram with a lognormal distribution we estimated the mean diameter value to be <*d*> = 9.19 nm (FWHM = 0.65 nm). Concerning the 6 nm and the 14 nm samples, the size has been evaluated from 25 different NPs in different areas. These images show that the density of NPs in the samples is clearly decreasing with increasing the NPs diameter. This is because for every sample we deposited an amount of NPs in order to have the same nominal film thickness of 10 nm. In Figure 2d a STM image of the non-epitaxial film is shown; the substrate is completely covered with a disordered film with a structured surface showing grains of a few nanometers in lateral size and a few angstroms in average height. After annealing the film up to *T* = 1040 K in O_2_ we obtained an epitaxial ceria thin film as shown in Figure 2e, in which the islands have atomically flat surfaces. In this case 45% of the Pt(111) substrate is covered and the islands have a mean height of 0.6 nm and a lateral size of 20–30 nm.

**Figure 3:**
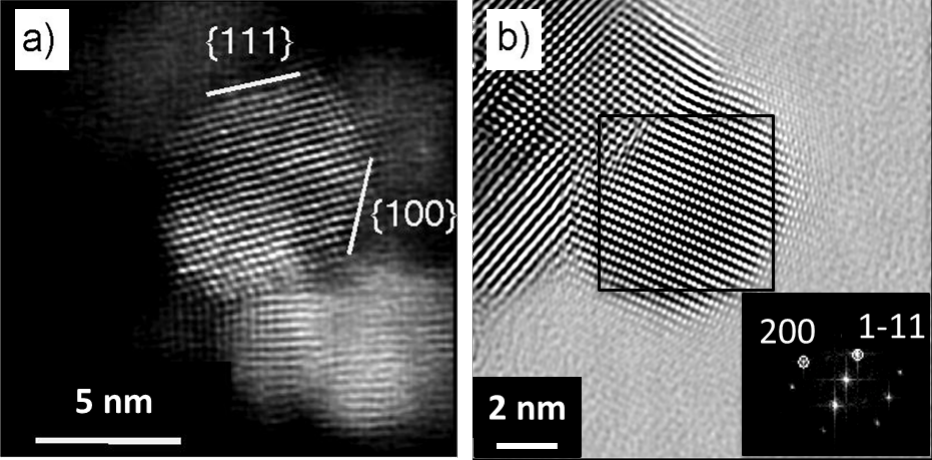
STEM (a) and HRTEM (b) images of ceria NPs corresponding to the sample with average diameter of 9 nm, the inset in (b) shows the FFT of the selected area with indicated the corresponding planes.

To gain a deeper insight into the atomic structure of the NPs, STEM and HR TEM measurements have been performed on 9 nm NPs deposited on a Lacey support grid, as shown in Figure 3a and Figure 3b respectively. Ce lattice fringes are also clearly visible. The NPs on the sample exhibit single crystalline structure (cubic CeO_2_, space group 225, *Fm−*3*m*), exposing frequently {111}, {220} and {100} facets, as evidenced in Figure 3a and Figure 3b. The (111) surface is indeed the most stable for cerium dioxide [1] and the (220) has the next lowest surface energy; at variance, the (100) surface is not as stable as the (111), but it is the most frequently exposed plane in ceria NPs after the (111) [23] probably because of the low dimensionality effects.

**Figure 4:**
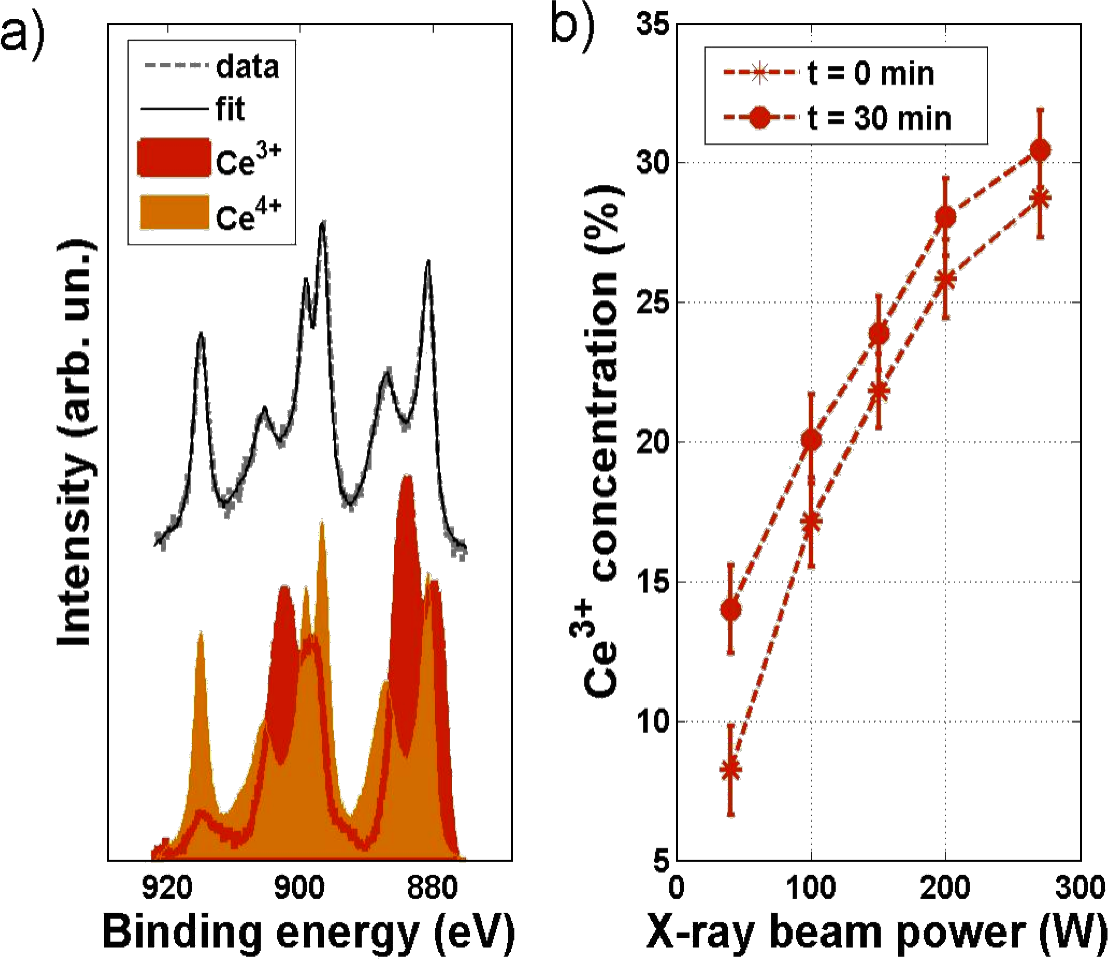
(a) Ce 3d XPS spectra and corresponding fit of the 9 nm sample acquired at 40 W, in the bottom region Ce^3+^ and Ce^4+^ XPS spectra**,** respectively**,** measured on NPs grown without oxygen in the system and then annealed in UHV at 1020 K, and on NPs oxidized directly in the aggregation chamber and then annealed in O_2_ at 1020 K**;** (b) Ce^3+^ concentration at different X-ray power acquired on the sample at the beginning of the acquisition for each X-ray power, *t* = 0 min, (red circle) and after 30 min (red star) of X-ray exposure, *t* = 30 min.

In Figure 4a a typical Ce 3d XPS spectrum of non-stoichiometric ceria NPs, with <*d*> = 6 nm, after annealing in O_2_ at *T* = 1020 K, is shown. In the spectrum it is possible to observe features from both Ce^3+^ and Ce^4+^ ionic species, as already observed for ceria NPs. In fact a core–shell model was proposed [24–25] for the oxidation state of the CeO_2−_*_x_* NPs, which assumes that the core of the nanoparticle is composed of CeO_2_ while the shell is composed of one layer of Ce_2_O_3_. This model well agrees with the observation in NPs with different size and shape for every synthesis procedures.

In the same figure the Ce 3d Ce^3+^ and Ce^4+^ reference spectra are also shown. It can be observed that Ce^3+^ reference spectrum shows a minor trace of Ce^4+^ related feature: in fact the peak present at binding energy value *BE* = 915 eV (see Figure 4a bottom panel red curve) is related with Ce^4+^ ions contribution. Because of the presence of this small peak, it was not possible to obtain an absolute value of the Ce^3+^ concentration, but the variation in the amount of Ce^3+^ ions as a function of size and annealing temperature could be monitored. It is also important to observe that UHV conditions and exposure to X-ray can reduce the samples, and thus Ce^3+^ concentration from XPS analysis can be overestimated [26]. The fitting curve obtained by using Equation 1 is also shown in the same figure; it can be observed that it is in good agreement with the experimental data.

As mentioned before CeO_2−_*_x_* NPs can be reduced under X-ray irradiation [26]. We performed an XPS analysis of the 9 nm sample while increasing the X-ray power and observed a progressive reduction of the NPs, and we evaluated the change of the concentration of the Ce^3+^ ions by performing the previously described fitting procedure. For each acquisition, the sample was kept under the flux for 30 min, and the Ce 3d spectrum was acquired at the beginning and at the end of each exposure for every value of the X-ray source operating power, in order to detect if the longer exposure affected the oxidation state. Fit results are shown in Figure 4b. Five different power values have been used: 40 W, 100 W, 150 W, 200 W, 270 W, the last one being the one that was used conventionally to perform XPS analysis. It is possible to observe that for low X-ray power contribution to the spectra coming from Ce^3+^ ions is very small (*a* = 8%, Table 1), and that it increases with the X-ray power; in particular for 270 W a value *a =* 30% was obtained. This reduction under UHV conditions and X-ray exposure at increasing power was not observed for the epitaxial film. In spite of the difficulties in obtaining absolute values of the concentration of Ce^3+^, it was possible to monitor the behavior of NPs in reduction and oxidation conditions.

**Table 1:** Ce^3+^ XPS intensity resulting from the fitting of the XPS spectra acquired at different X-ray power after different irradiation times.

power (W)	Ce^3+^ XPS intensity (%)	
	*t* = 0 min	*t* = 30 min

40	8.24 ± 1.59	14 ± 1.59
100	17.14 ± 1.59	20.09 ± 1.59
150	21.81 ± 1.32	23.93 ± 1.32
200	25.83 ± 1.39	28.05 ± 1.39
270	28.73 ± 1.39	30.48 ± 1.39

**Figure 5:**
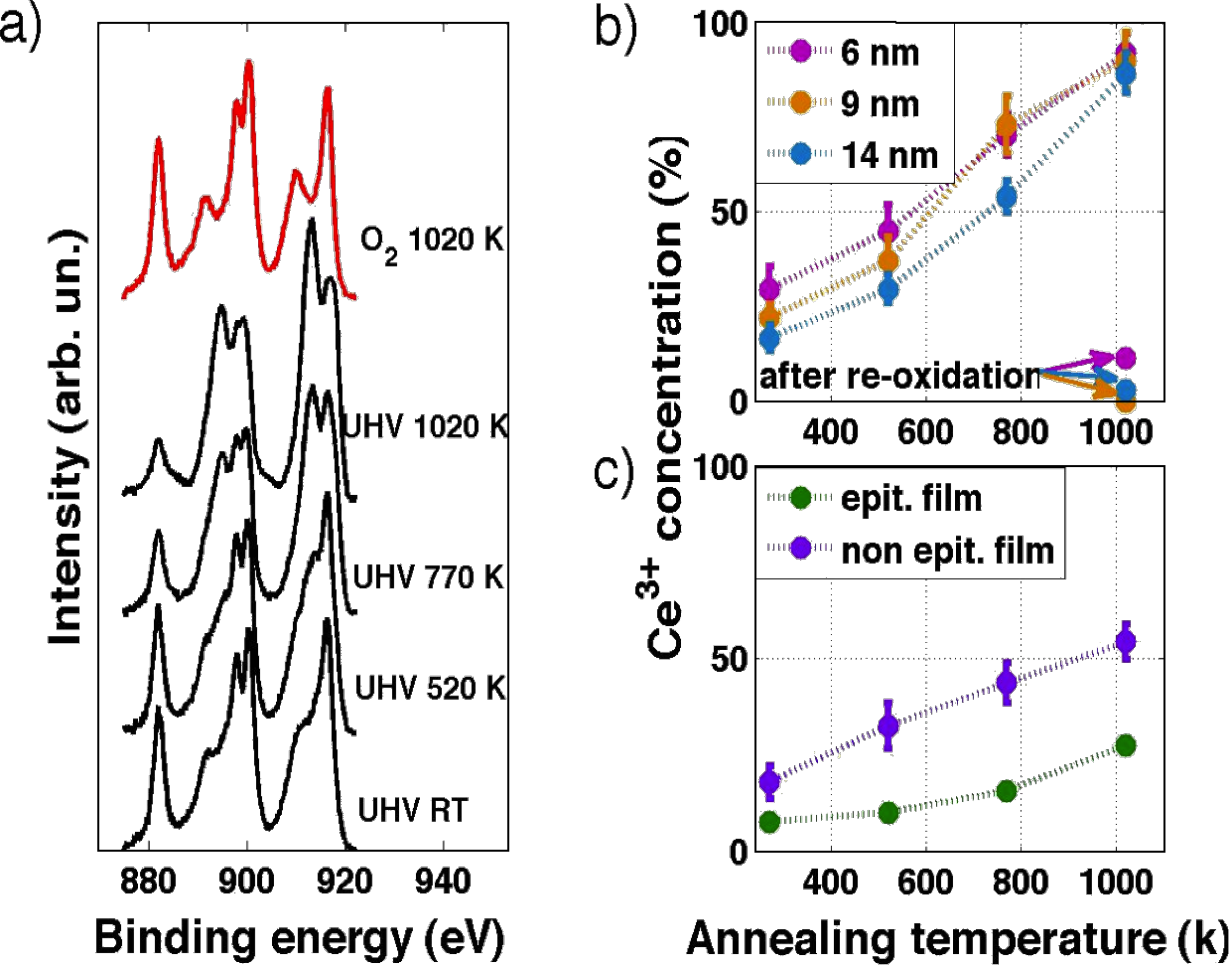
(a) Ce 3d XPS spectra for a complete reduction (black curves) and oxidation (red curve) cycle. (b) Intensity of the Ce^3+^ component at different annealing temperature and after re-oxidation on NPs of diameter of 6 nm (pink dots), 9 nm (orange dots), and 14 nm (blue dots), and (c) on the ultra-thin epitaxial (green dots) and non-epitaxial (violet dots) ceria film.

In Figure 5a, Ce 3d spectra for a complete reduction and oxidation cycle are shown. One can observe that the Ce^3+^-related features become more evident at increasing annealing temperatures. Performing the previously described fitting procedure, we monitored the intensity of the Ce^3+^ component in the XPS data as a function of the annealing temperature for all samples. Fit results are reported in Table 2, and they are plotted in Figure 5b for the NPs and in Figure 5c for the ultra-thin films.

**Table 2:** Ce^3+^ XPS intensity resulting from the fitting of the XPS spectra acquired at different annealing temperatures for NPs with different size and ultra-thin films under reduction and oxidation conditions.

annealing temperature (K)	Ce^3+^ XPS intensity (%)				
	6 nm	9 nm	14 nm	epitaxial film	non-epitaxial film

RT (UHV)	29.49 ± 5.87	21.84 ± 3.80	16.6 ± 3.35	7.57 ± 0.80	17.90 ± 4.33
520 (UHV)	44.82 ± 6.89	37.16 ± 6.12	29.57 ± 3.78	10.07 ± 1.23	32.47 ± 3.91
770 (UHV)	70.13 ± 5.43	72.89 ± 7.60	53.86 ± 4.17	15.64 ± 1.23	43.70 ± 5.21
1020 (UHV)	91.97 ± 5.12	89.95 ± 7.60	86.62 ± 5.35	27.37 ± 1.23	54.36 ± 4.62
1020 (O_2_)	11.31 ± 0.23	0 ± 0.25	2.87 ± 0.40		

The intensity of the Ce^3+^ component strongly depends on the NP size at room temperature (RT) and after thermal treatments at temperatures up to 770 K: NPs with average sizes of <*d*> = 6 nm and <*d*> = 14 nm present, respectively, the highest and lowest values of Ce^3+^ intensity, corresponding to the highest and lowest Ce^3+^ concentration, while 9 nm NPs with <*d*> = 9 nm exhibit an intermediate value. The reason for this behavior can be ascribed to the strong difference in the surface to volume ratio and to the oxygen vacancy energy formation [27]. After a thermal treatment at 1020 K, these differences in the Ce^3+^ component intensities are less significant; in agreement with the results reported in [27], the oxygen vacancy formation energy is related with NPs size, in particular it decreases with increasing the NPs size. It seems that the dimensionality has a strong contribution up to *T* = 770 K while at *T* = 1020 K the Ce^3+^ concentration is not related to the size.

**Figure 6:**
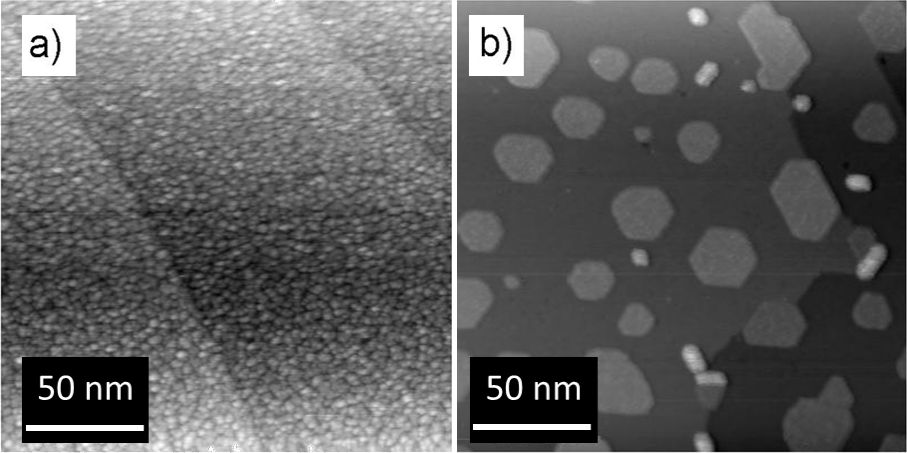
STM images of a cerium oxide ultrathin film on Pt(111) (a) as grown, acquired at 2.0 V and 0.03 nA, and (b) after annealing in UHV at 1020 K, acquired at 2.5 V and 0.03 nA.

In contrast, XPS from epitaxial film shows a very small concentration of Ce^3+^ ions already at RT, which increases with thermal treatments in vacuum. Moreover, the maximum value obtained for the intensity of Ce^3+^ component after thermal treatment at *T* = 1020 K is significantly lower with respect to the other samples (NPs and non-epitaxial film). Since the film has been chosen to have a comparable surface to volume ratio as the 9 nm NP, the lower degree of reduction is possibly due to the fact that the film exposes mainly (111) surfaces, which are the most stable ones. The non-epitaxial film instead shows a behavior closer to the NPs for thermal treatments up to *T* = 520 K, while for higher temperatures the Ce^3+^ concentration is lower than in the NPs. This significant difference can be ascribed to structural and morphological changes occurring in the non-epitaxial film at increasing temperature, as shown in Figure 6 in which STM images of the non-epitaxial film before and after the reduction cycle are reported. It is possible to observe that the as-grown film (Figure 6a) completely covers the substrate as a granular ultra-thin film (as in Figure 2d). After annealing in UHV at 1020 K it is possible to observe that the film becomes non-continuous with the formation of single quasi-hexagonal islands that cover almost 40% of the Pt(111) surface. So, because of the UHV annealing, the previously disordered ultra-thin film arranges in an ordered one exposing mainly the (111) surface, in analogy with previous studies investigating the changes of morphology after annealing the granular films in O_2_ [21].

## Conclusion

Non-stoichiometric ceria NPs have been synthetized through magnetron sputtering with a gas aggregation source. With a quadrupole filter NPs have been mass-selected obtaining a narrow size distribution, and three different lateral sizes have been selected. The morphology and stoichiometry of the NPs have been investigated and it was demonstrated that the concentration of Ce^3+^ ions decreases as a function of particle size for this kind of synthesis method. We investigated in detail how the combination of X-ray power, exposure and UHV conditions influence the oxidation state of the NPs and observed a partial reduction of the NPs. The variation of the Ce^3+^ concentration with thermal treatments was monitored with XPS, performing an analysis of the line shape of the Ce 3d spectrum. The oxidation state stability after thermal treatments in vacuum and in oxygen atmosphere has been studied for different particle sizes, and it has been compared with the epitaxial and non-epitaxial films. In this way the easier oxygen release in NPs synthetized by sputtering technique with respect to the films has been demonstrated. Such reducibility could affect the catalytic properties of ceria NPs.
